# Supplementary material for: Electrical Stimulation Induces Retinal Müller Cell Proliferation and Their Progenitor Cell Potential
Source: Cells. 2020 Mar 23;9(3):781. doi: 10.3390/cells9030781 (PMC7140850; doi:10.3390/cells9030781)
Supplement: Supplementary file 1 [file cells-09-00781-s001.pdf]

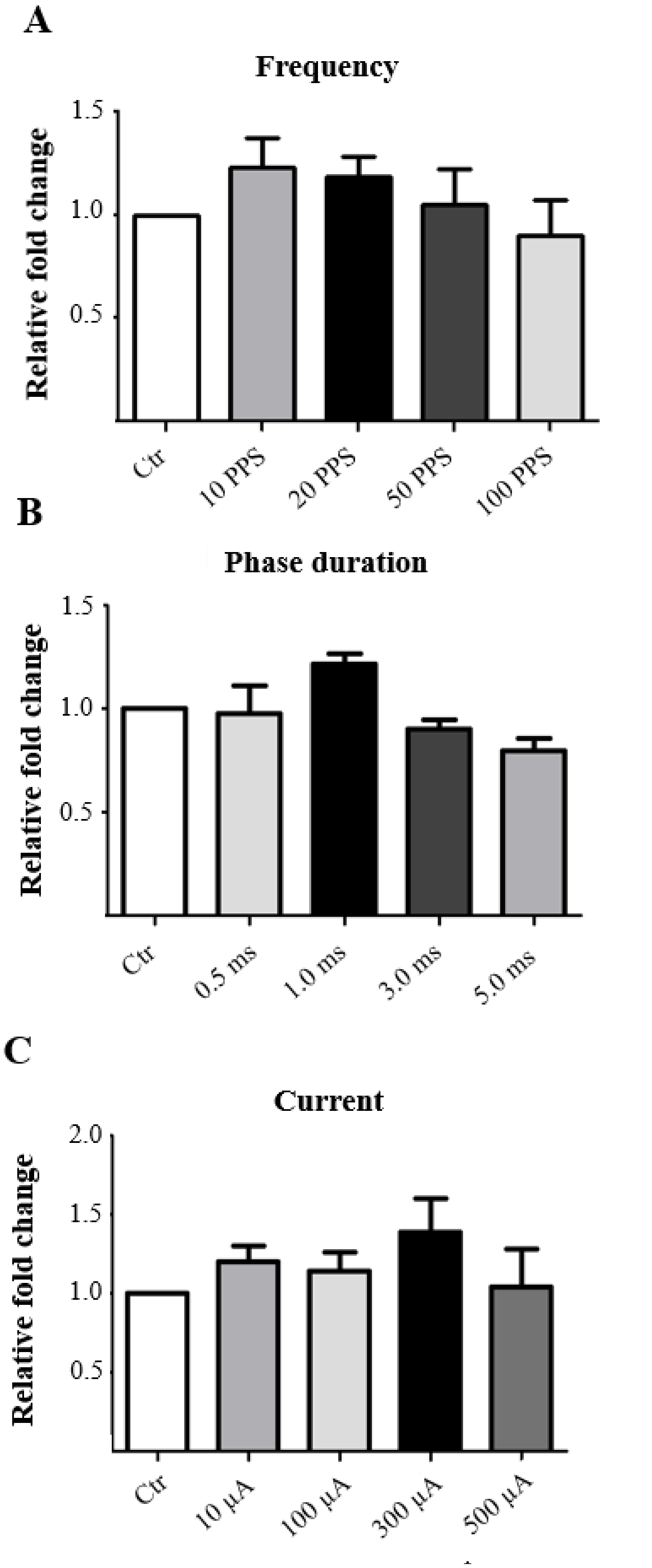
A
Frequency
Relative fold change
1.5
1.0
0.5
Ctr
10 PPS
20 PPS
50 PPS
100 PPS
B
Phase duration
Relative fold change
1.5
1.0
0.5
Ctr
0.5 ms
1.0 ms
3.0 ms
5.0 ms
C
Current
Relative fold change
2.0
1.5
1.0
0.5
Ctr
10 μA
100 μA
300 μA
500 μA

Figure S1. Impacts of ES frequency, duration, and current intensity on MC proliferation. **(A)** Relative changes in MC proliferation assessed by EdU incorporation assay, showing no significant differences under various PPS of ES at a rectangular waveform, 1 ms/phase duration, and 100 μA current. **(B)** Changes in MC proliferation under various phase durations of ES with a fixed rectangular waveform, 20 PPS, and 100 μA. **(C)** Changes in MC proliferation under various ES current with a rectangular waveform at 20 PPS and 1 ms phase duration. Value = means ± S.E.M. (n = 5/group compared to controls by paired *t* tests).

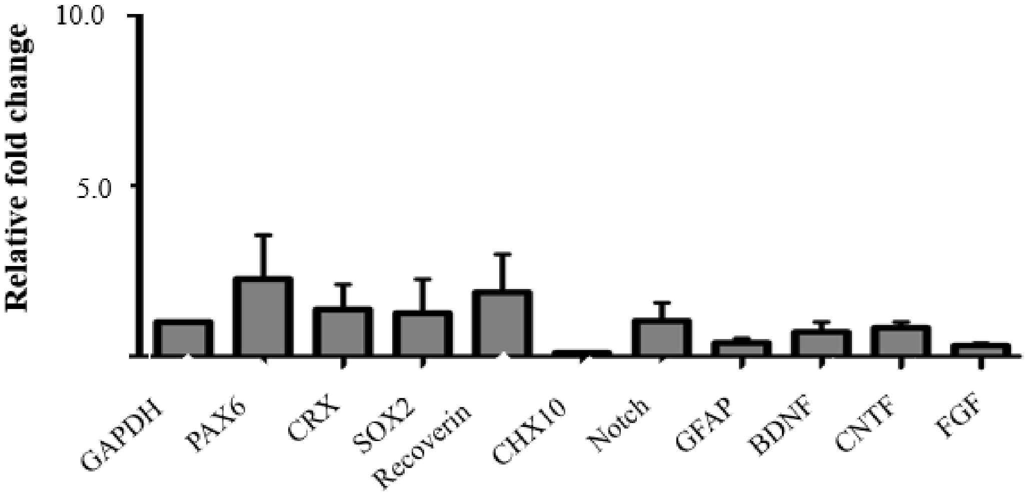

Figure S2. Impacts of ES frequency, duration and current intensity on MC proliferation. Relative fold changes of RT-PCR in progenitor cell markers after 15-minute of ES. Upregulation of several genes 48 hours after 15-minute ES treatment. The changes were non-significant.
